# An Ethnobotanical, Phytochemical Analysis, Antimicrobial and Biological Studies of *Pulicaria crispa* as a Graze Promising Shrub

**DOI:** 10.3390/life13112197

**Published:** 2023-11-11

**Authors:** Mashail N. AlZain, Fawziah M. Albarakaty, Rehab M. A. El-Desoukey

**Affiliations:** 1 Department of Biology, College of Sciences, Princess Nourah Bint Abdulrahman University, Riyadh 11451, Saudi Arabia; mnalzain@pnu.edu.sa; 2 Department of Biology, Faculty of Applied Science, Umm Al-Qura University, Makkah Al Mukarramah P.O. Box 715, Saudi Arabia; fmbarakati@uqu.edu.sa; 3 Microbiology and Immunology Department, National Research Centre, Giza 12622, Egypt; 4 Natural and Applied Sciences Department, Faculty of Science and Humanities in Afif, Shaqraa University, Shaqraa 11961, Saudi Arabia

**Keywords:** antimicrobial, *Pulicaria crispa*, gethgath, grazing herbs, antioxidant, animal pathogen, anticancer, ethnobotanical, antibiotic resistance

## Abstract

Due to the global issue of antimicrobial resistance, one of the most significant challenges in microbiological research is to develop a replacement antibiotic with minimal adverse effects. The wild shrub *Pulicaria crispa* (gethgath) has been traditionally used for camel and ruminant grazing. While prior research has demonstrated its antimicrobial properties against human diseases, no investigations have been conducted on its efficacy against animal pathogens. The objective of this study is to explore the ethnobotanical, phytochemical, antioxidant, anticancer, and antimicrobial activity of *Pulicaria crispa* aqueous and solvent extracts against a range of standard and animal pathogens. All of the extracts demonstrated antimicrobial, antioxidant, and anticancer properties, containing bioactive compounds. Notably, the ethyl acetate extract of *P. crispa* exhibited the strongest antimicrobial activity against tested Gram-positive and Gram-negative bacteria and fungi. The chloroform fraction displayed the highest antioxidant activity. Additionally, the ethyl acetate fraction showed promising anticancer activity against breast (MCF-7) and lung (A549) cancer cells. These findings confirm that *Pulicaria crispa* is a valuable shrub with potential applications as a natural alternative for antimicrobial, antioxidant, and anticancer treatments in both human and veterinary medicine.

## 1. Introduction

Infectious diseases have resurfaced as a significant global threat, resulting in substantial morbidity and mortality rates due to the occurrence of epidemics and pandemics [1]. This resurgence can be primarily attributed to the crisis of antimicrobial resistance (AMR) and the emergence of new and re-emerging viruses, particularly in the absence of preventive vaccines and effective antibiotics and antiviral drugs [1,2]. The improper or excessive utilization of antibiotics, coupled with the dearth of new drug discoveries, has facilitated the rapid proliferation of AMR [3]. Antibiotics play a critical role in preserving lives and enabling modern medical practices such as chemotherapy, organ transplantation, and surgery [4]. Consequently, there is an urgent need for the development of innovative antibacterial and antiviral agents that exhibit high efficacy, safety, and affordability against severe infectious diseases. Throughout history, natural products derived from plants, bacteria, fungi, and animals have been utilized as valuable sources of drugs or drug lead compounds, particularly for antimicrobial and antiviral purposes. This suggests that a return to nature could potentially alleviate the crisis surrounding infectious diseases. Notably, natural product drugs such as penicillin, cephalosporins, doxorubicin, aminoglycosides, amphenicols, tetracyclines, macrolides, and artemisinin have revolutionized the field of medicine [5].

**Figure 1:**
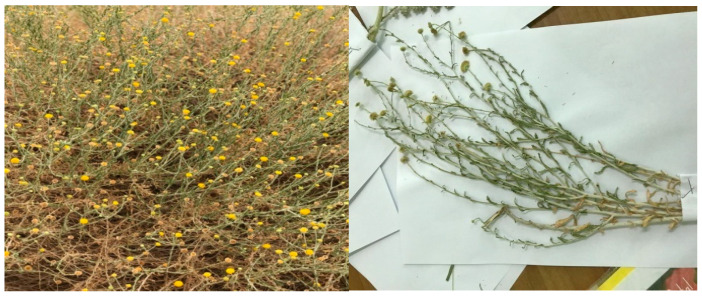
*Pulicaria crispa* (gethgath).

*Pulicaria crispa*, commonly known as gethgath (Figure 1), is a plant species belonging to the Asteraceae family. It is an annual herb, although it can sometimes be perennial, and produces small bright yellow flowers. This species is widely distributed and thrives in various regions including Pakistan, India, Saudi Arabia, Iran, Iraq, Kuwait, Afghanistan, and parts of North and West tropical Africa [6]. *Pulicaria crispa* has been traditionally used by Saudis and Egyptians for its medicinal properties. It is known to possess anti-inflammatory and insect-repelling properties and is often consumed as an herbal tea [7]. Medicinal herbs have long been recognized as valuable sources of secondary metabolites with therapeutic benefits [8]. Research data have shown that a significant proportion of new antibacterial agents introduced between the early 1980s and mid-2000s were derived from plants [9]. Additionally, approximately 50% of all anticancer agents developed between 1940 and 2014 were either extracted from plants or derived from their secondary metabolites [10]. The increasing demand for alternative treatments for various diseases, driven by factors such as resistance to cancer chemotherapy, multi-drug-resistant bacteria, adverse side effects of pharmacological therapies, and economic constraints, has led to a heightened interest in natural product chemistry research [11]. Simultaneously, the global burden of cancer is escalating at an alarming rate. Current projections indicate that the number of new cancer cases per year is expected to reach 21 million by 2030, resulting in approximately 17 million cancer-related deaths, with 75 million individuals living with cancer [12]. Natural products derived from plants, such as *Pulicaria crispa*, have demonstrated a wide range of biological properties including cytotoxic, anti-inflammatory, antimicrobial, and antioxidant effects [13]. Previous studies have identified several active ingredients in *Pulicaria crispa*. These include sterols like -sitosterol, stigmasterol, and lupeol; terpenes such as phytol; alkaloids like pulicarine and pulicaridine; flavonoids including quercetin, kaempferol, and luteolin; and phenols such as caffeic acid, chlorogenic acid, and gallic acid. These compounds have been associated with a range of biological activities, including antibacterial, antifungal, antiviral, antioxidant, anticancer, and anti-inflammatory effects [14,15]. To date, there has been a lack of research investigating the antimicrobial properties of an *P. crispa* extract against animal-sourced pathogens, despite its use as a grazing herb that is instinctively consumed by camels and small ruminants for its potential medicinal benefits. Zoopharmacognosy suggests that animals self-medicate by selecting natural substances, such as plants, herbs, clay, and insects, to mitigate the harmful effects of pathogens [16]. Furthermore, the safety of a *P. crispa* extract for therapeutic purposes has been established, with no significant toxicity reported [17]. Therefore, this study aims to investigate the phytochemical, ethnobotanical, antioxidant, anticancer, and antimicrobial effects of *P. crispa* against animal-sourced pathogens, with the goal of bringing greater attention to this overlooked shrub.

## 2. Materials and Methods

**Materials and Reagents:** All solvents and standards utilized in this study were of an analytical grade and were procured from Sigma-Aldrich. The experimental apparatus employed included a rotary evaporator (BȔCHI Rota-vapor R205, Flawil, Switzerland) and an HPTLC system (Eike Reich/CAMAG–Laboratory, Basel, Switzerland). The thin-layer chromatography (TLC) aluminum plates used were pre-coated with silica gel 60 F 254, measuring 10 × 5 cm and with a thickness of 0.2 mm, and were obtained from E. Merck Ltd., Mumbai, India.

**Collection of Shrub:** The aerial parts of *Pulicaria crispa* were gathered in April 2022, during the flowering season, from the Valley of the Quwai’ located in Al Quwai’iyah, a significant province adjacent to Riyadh Province in Saudi Arabia. The Province of Al-Quway’iyah, located in the Kingdom of Saudi Arabia, is renowned for its vast size. Situated on a flat plain, it is encompassed by mountain ranges on three sides—the north, west, and south. To the west lies the renowned supply chain of fiery configuration, while the eastern desert stretches out in a flat and wide sedimentary form. This desert, known as the Hadba desert, along with the desert Jala, represents the pinnacle of the Arabian Shield rock. Al-Quway’iyah occupies a significant position, serving as the meeting point between the ancient pyramids and the calcareous limestone rocks. Among its notable features is the Al Quwai’ valley (Wadi Al Quwai’), which descends from the west to the east before flowing into the Hadba desert and spreading throughout its expanse. The name Al-Quway’iyah is derived from this very valley. The valley is abundant with wild herbs and shrubs, which are utilized for animal grazing, fuel, and traditional medicine. Ethnobotanical inspections have revealed the presence of numerous traditional plants, many of which remain uncultivated or overlooked in various countries. Despite their significant local or global applications, these plants often go unnoticed or neglected. It is worth noting that these plants have demonstrated remarkable adaptability to challenging growth conditions, such as drought or salty environments. They contribute to nutrition and play a crucial role in naturalistic curative therapies [18].

**Bacterial and Fungal Strains:** The bacterial and fungal strains utilized in this research, namely *Enterococcus faecium*, *Bacillus Cereus*, *Klebsiella pneumoniae*, *Staphylococcus aureus*, *Escherichia coli*, *Streptococcus pyogens*, *Pseudomonas aerogenes*, *Salmonella typhimurium*, *and Candida albicans*, were obtained as animal pathogenic isolates. Additionally, standard strains including *Staph aureus ATCC 29213*, *Enterococcus faecalis ATCC 29212*, *E. coli ATCC 25922*, *S. typhimurium ATCC 14028*, *C. albicans ATCC 60193*, *C. tropicalis ATCC 66029*, and *C. tropicalis ATCC 66029* were procured from Sigma-Aldrich, Taufkirchen, Germany.

**Ethnobotanical Description:** Dr. Mashail N. AlZain, an Assistant Professor of Plant Ecology in the Department of Biology at the College of Sciences, Princess Nourah bint Abdulrahman University, conducted a thorough description and taxonomy of the shrub. This description was compared to the works of the authors of [19,20].

**Traditional and Therapeutic Uses of Shrubs:** The sources utilized in this study encompassed a comprehensive range of materials, including previous studies, textbooks, websites, journals, symposia, periodicals, and databases. These sources were specifically focused on the management of medicinal plants employed for the treatment of human diseases in Saudi Arabia, the Arabian Peninsula, and various other regions across the globe. It is worth noting that the accuracy and reliability of English/Arabic and Arabic/English dictionaries were verified and confirmed through the work of Hanan et al. (2019) [21].

**Aqueous Extraction:** The aerial components of the *P. crispa* shrub underwent a thorough washing process with clean distilled water, followed by air drying in a shaded area. Subsequently, the dried components were ground into a fine powder using a blender. Cold aqueous extracts were prepared using the infusion method as described in [22]. The preparation of hot aqueous extracts was carried out through the decoction method, as outlined in reference [22]. The extracts were then carefully stored at a temperature of 4 °C until they were needed.

**Solvent Extraction:** A total of 200 g of air-dried leaves was pulverized into a coarse powder. The powder was then soaked in 500 mL of 80% methanol at room temperature and left to stand for 2–3 days. This process was repeated until the plant material was fully utilized, resulting in a crude methanol extract. The crude extract was subsequently filtered and concentrated using a rotary evaporator set at 45 rpm and 40 °C, resulting in a dry crude extract weighing 51 g. Next, 100 g of the dry crude extract was suspended in water and subjected to fractionation using n-hexane, chloroform, ethyl acetate, ether, acetone, and n-butanol solvents. Each organic solvent was used in three separate 200 milliliter extractions. The filtrates obtained from each extraction were then concentrated using a rotary evaporator set at 45 rpm and 40 °C. The yields of the dried fractions obtained from the n-hexane, chloroform, ethyl acetate, and n-butanol solvents were 2.1%, 4.7%, 2%, and 5.1%, respectively. The dried fractions were carefully collected and stored in vials at a temperature of −20 °C until further use [23].

**Total Phenolic Content:** The estimation of phenolic contents in crude extracts was conducted using the method of [23]. In brief, an aliquot sample of 100 µL (2 mg/mL) or gallic acid, a standard phenolic (31.25–1000 µg/mL), was mixed with 1.5 mL of distilled water and 100 µL of a Folin–Ciocalteu reagent. The mixture was allowed to stand for 8 min at room temperature, which was followed by 300 µL of sodium carbonate (20%) being added. After incubation, the reaction mixture was thoroughly mixed and allowed to stand for 30 min at room temperature in the dark. The absorbance of all sample solutions was measured at 765 nm using a spectrophotometer. The phenolic content is expressed as the gallic acid equivalent per gram.

**Determination of Total Flavonoid Content:** The determination of the total flavonoid content was conducted using the method described in [24], which involves the formation of a flavonoid–aluminum complex. A 1 mL aliquot of the sample extract (2 mg/mL) was combined with 1 mL of a 2% aluminum chloride solution. Following a 15 min incubation period at room temperature, the absorbance of the resulting reaction mixture was measured at 430 nm using a spectrophotometer. A calibration curve was constructed using quercetin (3.125–100 µg/mL) as a standard. The quantity of flavonoids present was expressed as quercetin equivalents (QEs) per gram.

**The Determination of Antioxidant Activity:** This determination, specifically ABTS radical cation scavenging activity, was conducted on the extract and fractions of *P. crispa* leaves. This assay was slightly modified from the method described in [23]. In summary, a mixture of aqueous solutions containing ABTS (7 mM) and potassium persulfate (2.45 mM) was prepared in a 1:1 ratio. The mixture was then incubated for 0.5 h and refrigerated for 24 h before being diluted with ethanol. Subsequently, about 50 µmL from each different concentration of the ABTS solution was combined with each sample and left in the dark for 1 hour. The antioxidant percentage activity of the *P. crispa* extract and fractions was determined by measuring the reduction in ABTS, which was optically detected at 734 nm. The formula used to calculate the % of radical scavenging activity is as follows: % of radical scavenging activity = {(Abs control − Abs sample)/Abs control} × 100.

**MTT Cell Proliferation Assay:** Cell viability was assessed using the MTT assay, following the methodology previously described in [24]. A549 (lung) and MCF-7 (breast) cancer cells were seeded at a density of 20,000 cells per well in 96-well plates and allowed to incubate for 24 h. Subsequently, the cells were exposed to varying concentrations of each plant extract (500, 250, 125, 62.5 µg/mL), with doxorubicin serving as the positive control and untreated cells as the control group. After 48 h, 10 μL of an MTT solution (5 mg/mL in PBS) was added to each well and incubated for an additional 4 h at 37 °C. Following incubation, the formazan crystals were solubilized in 100 μL of acidified isopropanol, and the absorbance at 570 nm was measured using a plate reader (BioTek, Winooski, VT, USA. The cytotoxicity was expressed as a percentage relative to the control group. The half inhibitory concentration (IC50) was determined using a dose response curve, and cell viability was calculated using the following equation: Cell viability (%) = mean absorbance (treated cells)/mean absorbance (control) × 100.

**Disc Diffusion Assay:** The antimicrobial action of *P. crispa* extracts was evaluated using the agar well diffusion method on Mueller Hinton Agar (MHA) plates. The test organisms were cultured in a nutrient broth and incubated at 37 °C for bacteria and 25 °C for fungi overnight until a turbidity of 0.5 McFarland standards was achieved, resulting in a final inoculum of 1.5 × 10^8^ CFU/mL. The MHA plates were then inoculated with a standardized microbial culture broth. Plant extracts at a concentration of 50 mg/mL were prepared in Dimethyl Sulfoxide (DMSO). Six wells with a diameter of 6 mm were made in the inoculated media using a sterile cork borer. Each well was full with 50 µL of the extracts. Ciprofloxacin at 10 µg was used as a positive control for the bacterial strains and Nystatin (30 µg) for fungal strains. The plates were left to incubate at room temperature for approximately 30 min, followed by further incubation at 37 °C (for bacteria) and 25 °C (for fungi) for 18–24 h. After incubation, the plates were examined for the presence of a clear zone around the well, indicating antimicrobial activity of the tested compounds. The zone of inhibition (ZOI) was determined in mm [23].

**The Determination of the Minimum Inhibitory Concentration (MIC):** This involved the inoculation of *P. crispa*-susceptible bacterial and fungal strains into sterile peptone water, followed by overnight incubation at 37 °C (for bacteria) and 25 °C (for fungi). Subsequently, sterile test tubes were prepared by adding 9 mL of sterile peptone water, 1 mL of various concentrations of extracts, and 0.3 mL of the culture of the studied strains. The control was prepared using only peptone water and the extract. The inoculated and control tubes for both bacteria and fungi were incubated at 37 °C and 25 °C for 24 and 48 h, respectively, before being checked for turbidity. The MIC was determined as the lowest concentration that produced no turbidity [25].

**The Minimum Bactericidal or Fungicidal Concentration (MBC/MFC):** This was determined by performing the following procedure. Following incubation, samples from the tubes used in the Minimum Inhibitory Concentration (MIC) test that did not exhibit turbidity were streaked onto solidified Nutrient Agar (for bacteria) and Potato Dextrose Agar (for fungi) plates using sterile cotton swabs. These plates were then incubated at temperatures of 37 °C and 25 °C, respectively. After 24 and 48 h of incubation, the MBC was determined as the lowest concentration of the extract that showed no growth on the plates, indicating its bactericidal or fungicidal activity [25].

**Statistical Analysis:** The ANOVA and Origin Pro 8.5 were utilized to calculate the average and standard deviation after conducting all tests thrice. The least significant difference (LSD) test was employed to determine the mean differences and compare average values at a significance level of *p* ≤ 0.05. The software SAS version 9.4 was utilized to examine the correlation type and relationship between variables based on the value of r.

## 3. Results

The botanical description, traditional applications, and therapeutic uses of *P. crispa* are outlined in Table 1, which reveals that this plant belongs to the Asteraceae family and is commonly known as forssk. *P. crispa* is widely recognized as a valuable shrub in folk medicine across various countries, serving both human and animal populations. Additionally, it is utilized as a grazing resource. Numerous studies have substantiated its potential as an antidiabetic, antioxidant, anti-inflammatory, anticancer, and antimicrobial agent against certain human pathogens.

**Total phenolic and flavonoid content:** The data presented in Table 2 demonstrate that the total phenolic content of a *P. crispa* crude extract is equivalent to 93.7 mg GAE/g of dry extract, while the flavonoid content of a *P. crispa* crude extract is equivalent to 10.4 mg QE/g of dry extract.

**Figure 2:**
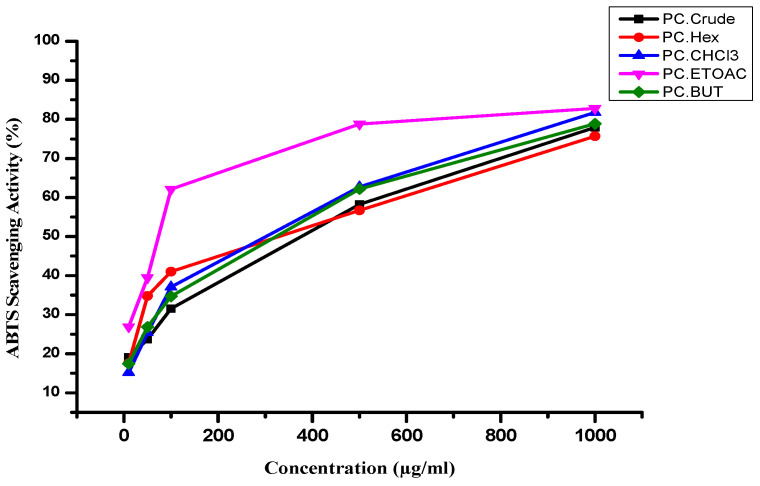
The antioxidant activity of *P. crispa* crude extract and fractions using ABTS scavenging activity method.

**Antioxidant scavenging activity**: The radical scavenging activity of the *P. crispa* extract and its fractions, as depicted in Figure 2, was evaluated using the ABTS assays. *P. crispa* demonstrated a remarkable capacity to scavenge free radicals at various concentrations. Notably, the chloroform fraction exhibited the highest antioxidant activity, with an IC50 value of 333.78 µg/mL. Conversely, the crude methanol extract displayed the weakest antioxidant activity, with an IC50 value of 490.14 µg/mL. The positive control quercetin IC50 value is 1.7 µg/mL.

**Figure 3:**
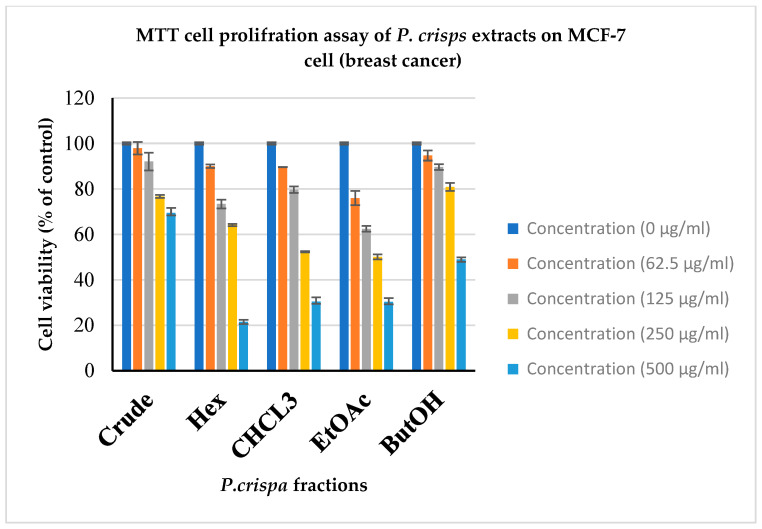
Antiproliferative effect of PC crude extract and its fractions on MCF-7 cell lines. Cells were treated with different concentration (0, 62.5, 125, 250, and 500 µg/mL) and cell viability inhibition is expressed as percentage (of negative control). Data represent the mean ± SD (standard deviation) of three independent experiments. Crude = crude methanol extract, Hex = hexane extract, CHCL3 = chloroform extract, EtOAc = ethyl acetate extract, ButOH = n-butane extract.

**Figure 4:**
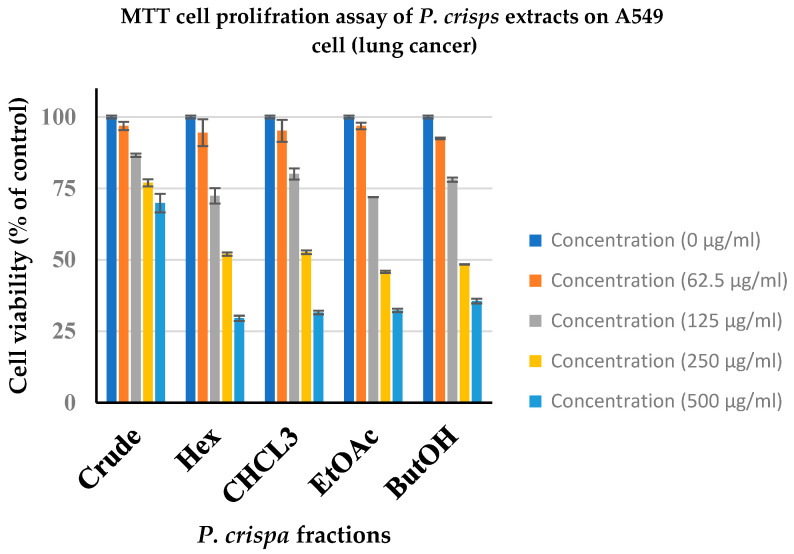
Antiproliferative effect of PC crude extract and its fractions on A549 cell lines. Cells were treated with different concentrations (0, 62.5, 125, 250, and 500 µg/mL) and cell viability inhibition is expressed as a percentage (of negative control). Data represent the mean ± SD (standard deviation) of three independent experiments. Crude = crude methanol extract, Hex = hexane extract, CHCL3 = chloroform extract, EtOAc = ethyl acetate extract, ButOH = n-butane extract.

**Cytotoxic activity**: The cytotoxic activity of the *P. crispa* extract and fractions was monitored with an in vitro cytotoxic MTT assay. The results of *P. crispa* cytotoxic activity against various cancer cells are summarized in Table 3. All analyzed fractions revealed a dose-dependent antiproliferative effect against all tested cancer cells (Figure 3 and Figure 4). Among all fractions tested, it was observed that the ethyl acetate fraction showed promising anticancer activity against breast (MCF-7) and lung (A549) cancer cells. The ethyl acetate showed the highest cytotoxic activity against both cancer cells (A549 and MCF-7) with IC50 values of 229.23 and 251.09 µg/mL, respectively. The other fractions displayed a weak inhibition toward a tested cancer cell line. The positive control was doxorubicin.

**Antimicrobial activity**: The extracts of the studied plant exhibited varying degrees of inhibition activity against the tested bacteria and fungi (Table 4 and Table 5), and the results were expressed in terms of the diameter of the growth-inhibition zone (clear zones). The results clearly showed that tested bacteria and fungi were susceptible to all extracts. There were significant differences (*p* < 0.05) in the mean diameter inhibition zone between the extracts. *P. crispa* ethyl acetate showed high activity against the examined bacteria and fungi strains followed by the methanol crude extract followed by the hot aqueous extract where n-hexane, n-butane, and ether possessed moderate activity against the examined strains. Meanwhile, the cold aqueous extract did not possess any antimicrobial effect against the examined strains.

### The Antimicrobial Assay, MIC, and MBC

Table 6, Table 7 and Table 8 demonstrate that all variants of shrub *P. crispa* extracts exhibit noteworthy antimicrobial activity against the animal pathogenic strains and standard strains that were examined. The data presented in Table 6, Table 7 and Table 8 indicate that the ethyl acetate extract displays the highest level of significant antimicrobial activity, followed by the crude methanol extract, and then the hot aqueous extracts. These findings, when combined with the results in Table 4 and Table 5, suggest that ethyl acetate and crude methanol extracts are the most effective solvents for producing *P. crispa* extracts, along with the hot aqueous extract, which possess potent antibacterial and antifungal properties.

## 4. Discussion

The discovery of new natural compounds is necessary to address the persistent global issues of antitumor and antimicrobial resistance. Bacterial and viral infections continue to pose a significant threat to global health, and the problem is worsened with antibiotic resistance and a lack of effective antiviral treatments [39]. Medicinal plants have become a hopeful avenue for finding new drugs, and *P. crispa* is a specific plant traditionally used in the Middle East for its various health benefits [40].

*Pulicaria crispa* (Forssk.) Oliv., a member of the *Asteraceae* family, has a wide range of medicinal properties that hold great potential. The *Asteraceae* family consists of around 100 genera and 2300 species, with Pulicaria being one of these genera, encompassing 100 species that are found all over the world. *P. crispa* (Forsk.) Oliv. (also known as *Francoeuria* (Forsk.)) is a wild aromatic plant commonly referred to as “gethgath”, which contains numerous compounds that are medically significant. This plant, which can be an annual herb or sometimes a perennial sub-shrub, produces small yellow flowers that contain essential oil with a strong aromatic scent. *P. crispa* is one of the most prevalent desert plants, growing naturally in Sudan, Saudi Arabia, Kuwait, Iran, Iraq, southern Egypt, Afghanistan, Pakistan, India, and parts of North and West tropical Africa. Various species of *Pulicaria* have traditionally been used in several countries to repel insects, alleviate back pain, treat intestinal disorders and inflammation, as well as reduce symptoms of influenza and the common cold; these species are also utilized in traditional veterinary medicine in Italy for their antiparasitic and repellent properties, as well as for treating respiratory ailments [41,42].

There is a lack of available literature regarding the antimicrobial impact of a *P. crispa* extract on animal pathogens. Consequently, this study represents pioneering research in this field, employing various aqueous and organic extracts. It is worth noting that previous studies have primarily focused on human pathogens, utilizing a limited range of extract types.

The objective of our study was to assess the phytochemical constituents, antimicrobial activity, and biological properties of both water and organic solvent extracts of *P. crispa*, with the aim of determining its potential as a valuable grazing shrub.

An extensive chemical analysis of *Pulicaria crispa* has revealed a wide range of phytoconstituents, including lipophilic, hydrophilic, and amphoteric substances [43]. *P. crispa* contains various classes of secondary metabolites, such as volatile oils, sterols, terpenes, flavonoids, glycosides, tannins, coumarins, and alkaloids, which contribute to its diverse pharmacological effects. Traditionally, *P. crispa* has been used for many years by the Sudanese, Egyptians, and Saudis to treat heart disorders, colic, coughs, colds, and excessive sweating. Additionally, *P. crispa* is known for its carminative, insect-repellent, and antimicrobial properties [44]. The chemical composition of the plant plays a crucial role in its potential as an alternative medicinal therapy, particularly due to its bioactive compounds such as alkaloids, flavonoids, tannins, and saponins [45]. Moreover, in developing countries, herbal remedies in folk medicine are trusted and utilized by many individuals for both humans and animals, as they offer a cost-effective and convenient treatment option [46].

The study presented in this paper reports on the total phenolic and flavonoid content of a *P. crispa* crude extract, which was found to be 93.7 mg GAE/g of dry extract and 10.4 mg QE/g of dry extract, respectively, as shown in Table 2. These results are consistent with previous research findings [38,46] and suggest that these two groups of phytochemicals may be responsible for the observed antimicrobial activity of *P. crispa* [47].

The literature contains several studies on the biological activity of *Pulicaria* species, including antimicrobial, antioxidant, anticholinesterase, analgesic, antipyretic, anti-inflammatory, cytotoxicity (HL-60, MCF-7, Hep-G2 cells), and hepatoprotective effects [31,32,33,34,35,36,37,38].

Consistent with these previous studies, the present study found that the *P. crispa* extract and fractions exhibited radical scavenging activity in the ABTS assays, with the chloroform fraction showing the highest antioxidant activity (IC50 = 333.78µg/mL) and the crude methanol extract showing the weakest antioxidant activity (IC50 = 490.14 µg/mL), as shown in Figure 2. The confirmation of these results can be validated, as indicated in the earlier investigation conducted in [48] where the absorbance of the DPPH radical is reduced by 66.193% when compared to the highest safe dose (EC100), demonstrating a significant antioxidant capacity. The scavenging activity of the DPPH radical is then quantified as “trolox equivalent antioxidant capacity” (TEAC), with a value of approximately 9.220086546 μg/mL. This implies that the oil’s highest percentage of scavenging activity is equivalent to the antioxidant potential of 9.22008654 μg/mL of trolox. Consequently, the essential oil of P. crispa can be considered a potent antioxidant agent. *P. crispa* exhibited robust antioxidant, antimicrobial, antifungal, and cytotoxic properties [47].

The cytotoxic activity of the extract and fractions of *P. crispa* was assessed using an in vitro cytotoxic MTT assay. The findings regarding the cytotoxic activity of *P. crispa* against various cancer cells are summarized in Table 2. It was observed that all fractions exhibited a dose-dependent antiproliferative effect on the tested cancer cells, as depicted in Figure 3 and Figure 4. Notably, the ethyl acetate fraction demonstrated significant anticancer activity against breast (MCF-7) and lung (A549) cancer cells. The ethyl acetate fraction exhibited the highest cytotoxic activity against both cancer cell lines (A549 and MCF-7), with IC50 values of 229.23 and 251.09 µg/mL, respectively. Conversely, the other fractions displayed limited inhibition toward the tested cancer cell line. The aforementioned agreement has been reached with regards to the numerical values [31,35,38,41]. This outcome substantiates the safety of the conventional applications of *P. crispa* for both human and animal consumption, as well as the demonstrated anticancer properties exhibited with the extracts of *P. crispa*.

Microbial pathogenesis often involves oxidative stress, as certain microorganisms produce reactive oxygen species (ROS) that can harm host cells and tissues. However, the use of antioxidant compounds can protect against oxidative damage and bolster the immune system response. Additionally, these compounds can influence the expression and activity of antimicrobial peptides, which are natural defense molecules that can disrupt microbial membranes or metabolism. Antioxidants can also work in tandem with conventional antibiotics, increasing their effectiveness while reducing toxicity. Furthermore, some antioxidants can even prevent the formation of biofilms, which are communities of microorganisms that are highly resistant to antibiotics. Thus, antimicrobial compounds with antioxidant properties offer a multifaceted approach to fighting infections and promoting overall human and animal health [49].

The present study investigated the potential antibacterial and antifungal activity of a crude extract (CME) and various fractions (HF, BF, EAF, AF, EF, and WF) derived from *P. crispa* against both Gram-positive and Gram-negative bacteria as well as fungi. The results showed that the EAF fraction had the strongest antibacterial effect, particularly against *K. pneumonia*, *S. aureus* (animal pathogenic isolate), and *Staph aureus ATCC 29213*, with a minimum inhibitory concentration (MIC) of 5 mg/mL for bacteria. This activity was even stronger than the reference standard used, ciprofloxacin. The EAF fraction also exhibited the strongest antifungal effect against *C. albicans* (animal pathogenic isolate), *C. albicans ATCC 60193*, and *C. tropicalis ATCC 66029*, with MIC values of 5 mg/mL and 25 mg/mL, respectively. This activity was even stronger than the reference standard used, Nystatin. These findings are consistent with recently published studies that reported the good antimicrobial properties of *P. crispa* against bacteria and fungi [34,36,41].

Further testing of the EAF fraction against additional Gram-positive and Gram-negative bacteria demonstrated promising antibacterial activity, with MIC values ranging from 25 to 75 mg/mL. Additionally, the minimum bactericidal concentrations (MBCs) were determined, with values ranging from 25 mg/mL to 100 mg/mL for each bacterial and fungal strain. This indicated the ability of the EAF fraction not only to inhibit bacterial growth but also to kill the bacteria. The MBC/MIC ratio, which is a valuable parameter in evaluating the effectiveness of antimicrobial agents, was calculated for the EAF, CM, and hot aqueous extracts against all bacteria and fungi tested. The results showed that the EAF, CM, and hot aqueous extracts exhibited a bactericidal and fungicidal effect against all tested bacteria, highlighting their potential as potent antimicrobial agents.

The findings of this study indicate that the extracts of *P. crispa*, including the ethyl acetate fraction (EAF), chloroform fraction (CM), and hot aqueous extract, have the potential to serve as a natural substitute for traditional antibiotics in the treatment of bacterial and fungal infections in animals. Additionally, the various fractions of *P. crispa*, namely the hexane fraction (HF), butanol fraction (BF), ethyl acetate fraction (EAF), and chloroform fraction (CHCL3), were evaluated for their cytotoxicity and potential antioxidant activity.

## 5. Conclusions

Plant products are currently generating significant interest as alternative medications. Our research has found that *P. crispa*, which grows in Egypt and Saudi Arabia, possesses exceptional properties such as anticancer, antioxidant, antifungal, and antibacterial effects. Specifically, the ethyl acetate fraction of *P. crispa* has demonstrated the most effective antimicrobial activity against both human and animal pathogens. This suggests that the *P. crispa* shrub, which naturally thrives in arid regions and can tolerate salty soil and limited water, holds great promise as a valuable grazing shrub. It provides animals with high-nutrition feed and serves as a natural prophylactic antimicrobial, eliminating the need for chemical antimicrobials in animal rations. This approach has the advantage of avoiding any side effects or residues in animal products, while also helping to reduce bacterial resistance in humans who consume these safe animal products. Furthermore, the ethyl acetate, methanol, and hot aqueous extracts have been identified as the most effective solvents for extracting bioactive compounds from the *P. crispa* shrub. These findings support and recommend the use of these solvents for future extraction processes.

## Figures and Tables

**Table 1:** This is a table demonstrating the ethnobotanical nature of *Pulicaria crispa*.

Ethnobotanical Items		References
Family name	*Asteraceae family*	[26]
Species name	*Pulicaria crispa*	[26]
Common name	Gethgath in Arabic: **جثجاث**	[26]
Plant life form	Shrublets	[26]
Favorable soil for plant	Sodium-rich soil, elevated terrain, and saline hollows.	[26,27]
Traditional and folk medicinal uses for humans	This medicinal plant has been utilized for numerous years in conventional medicine as an herbal tea for the treatment of heart diseases, as well as for its gastroprotective, anti-inflammatory, and insect-repellent properties. It is also commonly employed for alleviating back pain, inflammation, menstrual cramps, intestinal disorders, dysentery, and diarrhea. Additionally, it is known to be effective in treating colds, coughs, colic, excessive sweating, and carminative disorders. The aerial parts of this plant contain an essential oil with a potent aroma, which has been employed since ancient times in the traditional medicine system for treating sinusitis and respiratory tract infections.	[17,27,28]
Traditional and folk medicinal uses for animals	The plant is subject to grazing by domestic animals and camels. Additionally, these particular species are utilized in traditional Italian veterinary medicine as antiparasitics and repellents, and for the treatment of respiratory ailments.	[27,29,30]
Studies on plant	- Ethanol extracts derived from plants have demonstrated an anti-diabetic effect. - The ethanol extract of *P. crispa* exhibits a potent curative effect against CCl4-induced nephropathy by improving kidney function; reducing oxidative stress, inflammation, and nephrotoxicity index; and improving renal histopathological features. - Plant ethanolic extracts have been shown to possess antifibrotic, anti-inflammatory, and antioxidant effects in CCl4-induced liver fibrosis. - The healing potential of P. crispa in gastric ulcers may be attributed to its glycosides, coumarins, flavonoids, tannins, sterols, and triterpenes’ content. - Derivatives of P. crispa exhibit antioxidative and anti-inflammatory properties. - Plant extracts have demonstrated antibacterial effects against certain human pathogens, including Gram-negative bacteria and Candida albicans. - Plant extracts have shown antiparasitic effects against schistosoma. - These extracts possess various activities, such as anti-inflammatory, antileukemic, cancer chemo-preventive, and cytotoxic effects.	[17,31,32,33,34,35,36,37,38]

**Table 2:** Total phenolic and flavonoid content of *P. crispa* crude extract.

Plant	Total Phenol mg GAE/g of Dry Extract)	STD	Total Flavonoid mg QE/g of Dry Extract	STD
*P. crispa*	93.7	0.012	10.4	0.016

**Table 3:** IC50 values for the *P. crispa* crude extract and their fractions.

Cell Lines	PC Fractions’ IC_50_ (µg/ml)					C+ve
	Crude	Hex	CHCl_3_	EtoAc	ButOH	Doxorubicin
A549	NA	271.44 ± 3.3	278.95 ± 4.2	229.23 ± 2.72	241.68 ± 1.1	3.52 ± 0.05
MCF-7	NA	332.30 ± 0.5	273 ± 2.8	251.09 ± 1.94	488.97 ± 3.6	2.52 ± 0.03

NA = No Activity.

**Table 4:** The antimicrobial effect of *P. crispa* solvent extracts against standard strains.

Serotypes	G+ve		G-ve		Fungi	
Extracts	*S. aureus* *ATCC 29213*	*E. faecalis* *ATCC 29212*	*E. coli* *ATCC 25922*	*S. typhimurium* *ATCC 14028*	*C. albicans* *ATCC 60193*	*C. tropicalis* *ATCC 66029*
	Diameter of Inhibitory Zone in mm					
But	15.16 ± 0.15	11.66 ± 0.21	13.23 ± 0.25	11.43 ± 0.21	21.33 ± 0.15	22.23 ± 0.25
EtOAC	31.33 ± 0.21	18.26 ± 0.25	22.33 ± 0.21	19.67 ± 0.21	27.27 ± 0.25	24.13 ± 0.15
CHCL3	16.61 ± 0.30	17.61 ± 0.20	11.40 ± 0.20	15.56 ± 0.15	20.36 ± 0.15	21.72 ± 0.20
Hex	18.43 ± 0.25	15.06 ± 0.12	20.30 ± 0.20	16.70 ± 0.20	17.23 ± 0.25	25.33 ± 0.25
CM	20.70 ± 0.20	16.50 ± 0.10	21.50 ± 0.20	13.70 ± 0.20	24.40 ± 0.26	27.3 ± 0.26
E	18.66 ± 0.15	12.10 ± 0.10	18.40 ± 0.10	13.76 ± 0.15	16.46 ± 0.21	15.66 ± 0.21
A	15.26 ± 0.25	10.20 ± 0.20	12.30 ± 0.26	0	19.10 ± 0.10	21.16 ± 0.15
HA	30.13 ± 0.15	15.4 ± 0.26	16.30 ± 0.10	16.23 ± 0.15	23.5 ± 0.1	22.20 ± 0.20
CA	0	0	0	0	0	0
C-ve (D.W.)	0	0	0	0	0	0
C+ve/Cipro	22	18	20	18	NU	NU
C+ve/Ny	NU	NU	NU	NU	18	20

But = butanol, EtOAC = ethyl acetate, CHCL3 = chloroform, Hex = n-hexane, CM = crude methanol extract, A = acetone, E = ether, CA = cold aqueous extract, HA = hot aqueous extract, G+ve = Gram-positive bacteria, G-ve = Gram-negative bacteria, C+ve = positive control, C-ve = negative control, Cipro = ciprofloxacin (5 µg), Ny = Nystatin (30 µg), NU = not used. Ensure that the statistical analysis excludes both positive and negative controls.

**Table 5:** The antimicrobial effect of P. crispa extracts against isolated animal microbes.

Serotypes			Types of Extracts									C-ve	C+ve	C+ve
			Aqueous Extract		Organic Extracts							D.W.	Cipro	Ny
			HA	CA	Hex	But	EtOAC	E	A	CHCL3	CM			
G-ve	*Ps. aerogens*	Diameter of inhibitory zone in mm	29.33 ± 0.35	0	18.3 ± 0.14	22.33 ± 0.49	31.57 ± 0.51	14.25 ± 0.35	10.3 ± 0.26	27.2 ± 0.28	13.23 ± 0.25	0	31	NU
	*E. coli*		15.23 ± 0.32	0	19.3 ± 0.2	12.37 ± 0.35	20.27 ± 0.25	17.23 ± 0.25	11.55 ± 0.54	10.33 ± 0.42	18.33 ± 0.35	0	20	NU
	*Enterococcus*		14.4 ± 0.10	0	13.2 ± 0.26	10.27 ± 0.25	17.43 ± 0.40	11.37 ± 0.21	0	16.2 ± 0.26	15.13 ± 0.15	0	35	NU
	*Salmonella*		15.37 ± 0.38	0	15.27 ± 0.21	10.16 ± 0.15	18.33 ± 0.21	12.3 ± 0.26	0	13.27 ± 0.21	12.33 ± 0.31	0	30	NU
G+ve	*K. pneumonia*		24.27 ± 0.31	0	14.27 ± 0.23	18.1 ± 0.12	35.3 ± 0.36	12.2 ± 0.2	11.03 ± 0.1	30.3 ± 0.36	15.1 ± 0.10	0	34	NU
	*Bacillus*		19.2 ± 0.2	0	20.26 ± 0.21	16.73 ± 0.15	24.1 ± 0.10	20.3 ± 0.30	16.27 ± 0.21	15.2 ± 0.20	22.4 ± 0.10	0	34	NU
	*S. pyogens*		24.37 ± 0.35	0	18.33 ± 0.25	27.4 ± 0.20	29.53 ± 0.21	17.23 ± 0.25	25.3 ± 0.36	24.4 ± 0.40	26.23 ± 0.25	0	R	NU
	*S. aureus*		29.4 ± 0.20	0	17.17 ± 0.15	14.37 ± 0.32	30.73 ± 0.21	17.37 ± 0.35	14.27 ± 0.25	15.47 ± 0.45	19.03 ± 0.15	0	25	NU
Fungi	*C. albicans*		22.26 ± 0.25	0	16.53 ± 0.15	20.23 ± 0.25	26.8 ± 0.10	15.43 ± 0.25	18.4 ± 0.26	19.3 ± 0.30	25.2 ± 0.26	0	NU	16

But = butanol, EtOAC = ethyl acetate, CHCL3 = chloroform, Hex = n-hexane, CM = crude methanol extract, A = acetone, E = ether, CA = cold aqueous extract, HA = hot aqueous extract, G+ve = Gram-positive bacteria, G-ve = Gram-negative bacteria, C+ve = positive control, C-ve = negative control, Cipro = ciprofloxacin (5 µg), Ny = Nystatin (30 µg), NU = not used. Ensure that the statistical analysis excludes both positive and negative controls.

**Table 6:** This is a table demonstrating the MIC and MBC/MFC of the ethyl acetate extract of *Pulicaria crispa*.

Examined Strains	Concentrations of Ether Extract (mg/mL)							
	5	25	50	75	100	150	MIC	MBC/MFC
*Ps. aerogensa*	_	_	_	_	_	_	5 mg/mL	25 mg/mL
*E. coli*	+	_	_	_	_	_	25 mg/mL	50 mg/mL
*Enterococcus*	++	+	_	_	_	_	50 mg/mL	75 mg/mL
*Salmonella*	++	+	_	_	_	_	50 mg/mL	75 mg/mL
*K. pneumonia*	_	_	_	_	_	_	5 mg/mL	25 mg/mL
*Bacillus*	+	_	_	_	_	_	25 mg/mL	50 mg/mL
*S. pyogens*	_	_	_	_	_	_	5 mg/mL	25 mg/mL
*S. aureus*	_	_	_	_	_	_	5 mg/mL	25 mg/mL
*Candida albicans*	+	_	_	_	_	_	25 mg/mL	50 mg/mL
*Staph aureus ATCC 29213*	_	_	_	_	_	_	5 mg/mL	25 mg/mL
*Enterococcus faecalis ATCC 29212*	++	++	+	_	_	_	75 mg/mL	100 mg/mL
*E. coli ATCC 25922*	+	_	_	_	_	_	25 mg/mL	50 mg/mL
*S. typhimurium ATCC 14028*	++	++	+	_	_	_	75 mg/mL	100 mg/mL
*C. albicans ATCC 60193*	_	_	_	_	_	_	5 mg/mL	25 mg/mL
*C. tropicalis ATCC 6602*	+	_	_	_	_	_	25 mg/mL	50 mg/mL

(+) = turbid (microbial growth), (++) = very turbid (high microbial growth), (_) = no turbidity (no microbial growth).

**Table 7:** This is a table demonstrating the MIC and MBC/MFC of the crude methanol extract of *Pulicaria crispa*.

Examined Strains	Concentrations of Ether Extract (mg/mL)							
	5	25	50	75	100	150	MIC	MBC/MFC
*Ps. aerogensa*	++	++	+	_	_	_	75 mg/mL	100 mg/mL
*E. coli*	++	++	+	_	_	_	75 mg/mL	100 mg/mL
*Enterococcus*	++	++	+	_	_	_	75 mg/mL	100 mg/mL
*Salmonella*	++	++	+	_	_	_	75 mg/mL	100 mg/mL
*K. pneumonia*	++	++	+	_	_	_	75 mg/mL	100 mg/mL
*Bacillus*	++	+	_	_	_	_	50 mg/mL	75 mg/mL
*S. pyogens*	++	+	_	_	_	_	50 mg/mL	75 mg/mL
*S. aureus*	++	++	+	_	_	_	75 mg/mL	100 mg/mL
*Candida albicans*	++	+	_	_	_	_	50 mg/mL	75 mg/mL
*Staph aureus ATCC 29213*	++	+	_	_	_	_	50 mg/mL	75 mg/mL
*Enterococcus faecalis ATCC 29212*	++	++	+	_	_	_	75 mg/mL	100 mg/mL
*E. coli ATCC 25922*	++	+	_	_	_	_	50 mg/mL	75 mg/mL
*S. typhimurium ATCC 14028*	++	++	+	_	_	_	75 mg/mL	100 mg/mL
*C. albicans ATCC 60193*	++	+	_	_	_	_	50 mg/mL	75 mg/mL
*C. tropicalis ATCC 6602*	_	_	_	_	_	_	5 mg/mL	25 mg/mL

(+) = turbid (microbial growth), (++) = very turbid (high microbial growth), (_) = no turbidity (no microbial growth).

**Table 8:** This is a table demonstrating the MIC and MBC/MFC of the hot aqueous extract of *Pulicaria crispa*.

Examined Strains	Concentrations of Ether Extract (mg/mL)							
	5	25	50	75	100	150	MIC	MBC/MFC
*Ps. aerogensa*	_	_	_	_	_	_	5 mg/mL	25 mg/mL
*E. coli*	++	++	+	_	_	_	75 mg/mL	100 mg/mL
*Enterococcus*	++	++	+	_	_	_	75 mg/mL	100 mg/mL
*Salmonella*	++	++	+	_	_	_	75 mg/mL	100 mg/mL
*K. pneumonia*	++	+	_	_	_	_	50 mg/mL	75 mg/mL
*Bacillus*	++	++	+	_	_	_	75 mg/mL	100 mg/mL
*S. pyogens*	++	+	_	_	_	_	50 mg/mL	75 mg/mL
*S. aureus*	_	_	_	_	_	_	5 mg/mL	25 mg/mL
*Candida albicans*	++	+	_	_	_	_	50 mg/mL	75 mg/mL
*Staph aureus ATCC 29213*	_	_	_	_	_	_	5 mg/mL	25 mg/mL
*Enterococcus faecalis ATCC 29212*	++	++	+	_	_	_	75 mg/mL	100 mg/mL
*E. coli ATCC 25922*	++	++	+	_	_	_	75 mg/mL	100 mg/mL
*S. typhimurium ATCC 14028*	++	++	+	_	_	_	75 mg/mL	100 mg/mL
*C. albicans ATCC 60193*	++	+	_	_	_	_	50 mg/mL	75 mg/mL
*C. tropicalis ATCC 6602*	++	+	_	_	_	_	50 mg/mL	75 mg/mL

(+) = turbid (microbial growth), (++) = very turbid (high microbial growth), (_) = no turbidity (no microbial growth).

## Data Availability

Data is contained within the article.
